# The effects of preoperative supplementation with a combination of beta‐hydroxy‐beta‐methylbutyrate, arginine, and glutamine on inflammatory and hematological markers of patients with heart surgery: a randomized controlled trial

**DOI:** 10.1186/s12893-022-01495-1

**Published:** 2022-02-11

**Authors:** Mona Norouzi, Azadeh Nadjarzadeh, Majid Maleki, Sayyed Saeid Khayyatzadeh, Saeid Hosseini, Mehdi Yaseri, Hamed Fattahi

**Affiliations:** 1 Department of Nutrition, International Campus of Shahid Sadoughi University of Medical Science, Yazd, Iran; 2 Nutrition and Food Security Research Center, Shahid Sadoughi University of Medical Sciences, Yazd, Iran; 3 Department of Nutrition, School of Public Health, Shahid Sadoughi University of Medical Sciences, Yazd, Iran; 4 Rajaie Cardiovascular Medical and Research Center, Iran University of Medical Sciences, Tehran, Iran; 5 Heart Valve Disease Research Center, Shahid Rajaie Cardiovascular Medical and Research Center, Iran University of Medical Sciences, Tehran, Iran; 6 Department of Epidemiology and Biostatistics, Tehran University of Medical Science, Tehran, Iran; 7 Cardiovascular Medical and Research Center, Shahid Beheshti University of Medical Sciences, Tehran, Iran

**Keywords:** Beta-hydroxy-beta-methylbutyrate, Glutamine, HMB, Arginine, Heart surgery

## Abstract

**Background:**

Cardiac surgery is associated with a widespread inflammatory response, by an additional release of free radicals. Due to the importance of these patient’s nutritional status, the present study was designed to evaluate the effectiveness of supplementation with a combination of glutamine, β-hydroxy-β-methylbutyrate (HMB) and arginine in patients undergoing to the heart surgery.

**Methods:**

The experiment was performed in 1 month (30 days) before cardiac surgery. patients were asked to take 2 sachets of Heallagen® (a combination of 7 g l-arginine, 7 g l-glutamine, and 1.5 g daily HMB) or placebo with identical appearance and taste (maltodextrin) with 120 cc of water. Clinical and biochemical factors were evaluated in the baseline and end of the study.

**Results:**

Totally, 60 preoperative patients (30 interventions and 30 placeboes) with a mean age of 53.13 ± 14.35 years participated in the study. Subjects in Heallagen® group had a lower serum levels of interleukin-6 (P = 0.023), erythrocyte sedimentation rate (P < 0.01), high sensitivity C-reactive protein (P < 0.01), and lymphocyte number (P = 0.007) compared to the placebo, at end of the study.

**Conclusion:**

In the patients undergoing heart surgery, Heallagen® significantly improved some of the inflammatory factors and hematological parameters. These results need to be confirmed in a larger trial.

*Trial registration:* The protocol of the study was registered in the IRCT.ir with registration no. IRCT20120913010826N31 at 13/10/2020.

## Introduction

The increases in life expectancy as well as the high prevalence of cardiovascular disease in the elderly warranted the increase of heart surgery in recent years in most developed and developing countries [1, 2]. Surgical stress causes an acute systemic inflammatory response, which has been proven in numerous studies. The primary inflammatory response is built to eliminate germs, facilitate healing after injury, and recover homeostasis [3]. However, persistent or severe inflammatory reactions may exceed the host's compensating mechanisms, resulting in multiple-organ failure, and patient's death. Cytokines are key mediators of the inflammatory response. The reactions of some pro inflammatory cytokines such as tumor necrosis factor-alpha (TNF-α), interleukin-6 (IL-6), and interleukin-10 (IL-10) have all been characterized in the situation of sepsis, surgical damage, and trauma [4]. In cardiac surgery, a widespread inflammatory response, by an additional release of free radicals related to the surgical trauma, transfusion, hypothermia, and the extra corporeal circulation were identified [5]. Malnutrition is one of the most common consequences observed in patients with heart failure or candidates for heart surgery, which is strongly associated with prolonged recovery after surgery and increased mortality in patients with heart failure [6–8]. Previous studies have shown that poor nutritional status and sarcopenia in patients with heart failure delay the patient's recovery and increase incidence of complications in cardiac surgery [9, 10]. Sarcopenia and cachexia, which occurs frequently in patients with severe cardiovascular disease impairs the capability of reaction to disease or injury, worsens the prognosis of systemic inflammation, and increases morbidity and mortality [11].

Previous studies have demonstrated that effective and timely nutritional support in candidates of heart surgery can strongly prevents inflammatory cardiac diseases (such as myocarditis), decreases the nutritional deficiencies after surgery, accelerates the recovery process, and improves the prognosis of disease [12, 13]. It has been shown that immune-boosting supplements such as omega-3, l-arginine and vitamin D speed up recovery process and reduce serum concentration of pro inflammatory biomarkers [14–17]. Also, the amount and type of protein intake has been studied [13]; although some studies indicated that taking protein from diet or supplements is associated with the prevention of excessive inflammatory responses [18, 19], some others reported conflicting results [20, 21]. Compared to the total protein, taking supplements containing specific amino acids might be more effective in modulating the increase in the level of inflammatory factors [22, 23]. Certain amino acids such as glutamine (Gln) and β-hydroxy β-methylbutyric acid (HMB) have been shown to speed up healing process of diabetic foot ulcers in animal specimens [24, 25]. Also, it has been reported that HMB supplementation, which is a metabolite of leucine, may attenuate the pro-inflammatory response [26].

Glutamine and arginine (Arg) are two important amino acids, which may be conditionally essential under severe stressful conditions [27]. Gln administration were related to good nitrogen balance in patient after trauma surgery [28]. Also, it has been reported that Gln and Arg co-administration can decrease the production of proinflammatory cytokines [27]. Despite the role of special amino acids in the prevention of exacerbated inflammatory response, based on our knowledge, there is not high-quality clinical studies which evaluate the effects of special amino acids in the management of heart surgery inflammation. So, this study designed to evaluate the effects of co-supplementation with HMB, Arg, and Gln on inflammatory and hematological markers among the patients with heart Surgery.

## Materials and methods

### Study design and participants

A double-blind, randomized placebo-controlled trial was performed to evaluate the effect of a multi-ingredients dietary supplement of HMB, Arg, and Gln (Heallagen®) in 1 month before surgery on post-surgery outcomes. Totally, 70 subjects were recruited in parallel design with 1:1 ratio of allocation to the intervention and control groups from candidates of cardiac surgery referred to Shahid Rajaei educational, research, and medical center of cardiovascular diseases, Tehran, Iran. The sample size was calculated according to the 7.5 units change (δ) of CRP based on a pilot study on 50 patients before and after study using the following equation:

$$n=\frac{2\times {\left({Z}_{1-\frac{\alpha }{2}}+{Z}_{1-\beta }\right)}^{2}\times ({{2\sigma }_{diff}}^{2}) }{{\updelta }^{2}}$$

By considering 0.05 of probability of type I error (α), 95% of power (1-β), and 10% of drop-out, the sample size was determined as 35 subjects in each group. Cardiac surgery was defined by mitral valve replacement, aortic root surgery, aortic valve replacement, coronary artery bypass graft surgery, and different combinations of these surgical procedures. Inclusion criteria were defined as age between 18 to 70 years, body mass index (BMI) between 18.5 to 29.9 kg/m^2^, non-existence of infection and sepsis at the baseline, no need for other enteral formulas due to special conditions and willing to participate in the study. Individuals with liver or autoimmune diseases, metastatic cancer, abnormality in thyroid, pituitary and hypothalamus function and gastrointestinal diseases were not included to our study. Patients with receiving immunosuppressive drugs or drugs that affect metabolism, allergies, or intolerance to ingredients of the Heallagen® or placebo were excluded from our study. The present study was conducted in accordance with the deceleration of Helsinki and the Ethics Committee of Shahid Sadoughi University of Medical Sciences approved the protocol of the study (IR.55U.SPH.REC.1398.122). The protocol of the study was registered in the Iranian Registry of Clinical Trials with registration no. IRCT20120913010826N31 at 13/10/2020. All participants completed and signed a written informed consent.

### Randomization, blinding, and intervention

**Fig. 1:**
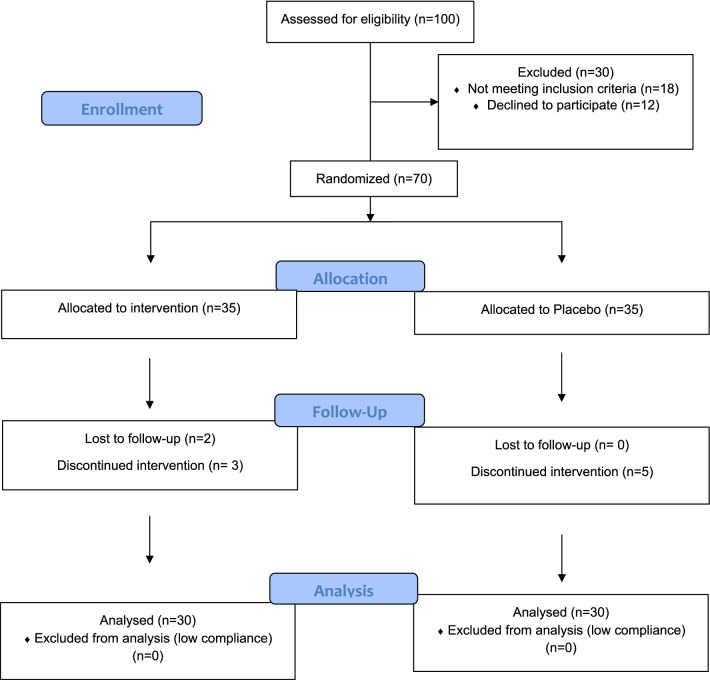
The CONSORT flow diagram of the study participants

The participant's enrollment flowchart is outlined in Fig. 1. The Blocked Randomization with block size equals to 2 was performed by an independent statistician. Someone blinded to aims of the study and patients baseline status opened sealed envelopes at the time of group assignments. Patients that met inclusion criteria were assigned to receive 2 sachets/day of Heallagen® (a combination of 7 g L-arginine, 7 g L-glutamine and 1.5 g HMB) or placebo with identical appearance and taste (maltodextrin) with 120 cc of water for 30 days before surgery. The Heallagen® and placebo were provided by Karen Pharma & Food Supplements company (Tehran, Iran). Participants, caregivers, and researchers assessing outcomes were blinded to the assignments.

### Procedures

Baseline measurements were performed in 1 month (30 days) before cardiac surgery. At the start of the study the data of age, gender, history of chronic disease, use of drugs or supplements, history of diagnosed hypertension (HTN) and smoking status were recorded. Anthropometric indices (weight and height) measured at the start and end of the study. Also, venous blood samples were collected at the baseline (30 days before surgery) and 1 day after surgery. At the time of assignment, 60 sachets were provided to each participant and they were asked to receive 2 sachets/day. Patients were requested to return the sachets on the day of their visit to the hospital for surgery to assess their compliance. Consuming less than 90% of the supplements or placebo were considered as low compliance and excluded from the study.

### Primary outcomes

Inflammatory markers were included the serum levels of interleukin-1 (IL-1), interleukin-6 (IL-6), tumor necrosis factor-alpha (TNF-α), high sensitivity C-reactive protein (hs-CRP), and erythrocyte sedimentation rate (ESR).

### Secondary outcomes

Secondary outcomes included the counts of red blood cells (RBC), white blood cells (WBC), platelet, neutrophil, and lymphocyte, serum levels of blood urea nitrogen (BUN), and creatinine.

### Biochemical assessments

Ten milliliters (ml) of antecubital venous blood samples were collected at the baseline and 1 day after surgery between 7:00 to 9:00 am. Five ml of Ethylenediaminetetraacetic acid (EDTA) anticoagulated blood sample was used to assess the counts of RBC, WBC, and Platelets. ESR was evaluated using the method recommended by International Council for Standardization in Haematology (ICSH) [29]. Five ml of blood samples were centrifuged for 10 min at room temperature with 4000 rpm to isolate the serum. These serums were stored at – 80 °C until biochemical analysis. Measurement of serum IL-6, IL-1, and TNF-α levels was performed using the enzyme-linked immunosorbent assay (ELISA) kits (Mabtag GmbH, Friesoythe, Germany). Serum concentrations of hs-CRP, BUN, and creatinine were assessed using Hitachi 917 autoanalyzer (Japan).

### Anthropometric measurements

A Seca scale was used to weigh patients with a precision of 100 g, with the minimal dress and without shoes. To assess their height with a precision of 1 mm, a Seca stadiometer was used in a standing position next to the wall and without shoes. If it was not possible to measure standing height, the length was assessed in a lying position. Dividing the weight (kg) by the square of height (m^2^) formula resulted in the BMI. Subjects with All measurements were repeated 3 times, and the mean of measurements was used to establish the re-test reliability.

### Statistical methods

Kolmogorov–Smirnov test was used to evaluate the normality of quantitative variables. Quantitative and qualitative variables were presented as mean ± SD and frequency (%), respectively. To compare quantitative data between two groups, independent sample t-test and Mann–Whitney test were applied for normal and non-normal variables, respectively. Within-group comparison was assessed using paired sample t-test for variables (or their log-transformed values) with a normal distribution and Wilcoxon test for non-normal distributed variables. To adjust the effect of confounding variables (BMI, age, gender, and history of HTN) the analysis of covariance (ANCOVA) or non-parametric ranked ANCOVA were used. Statistical analyses were conducted using SPSS software version 25 (IBM Corp. IBM SPSS Statistics for Windows, Armonk, NY) and RStudio software version 1.4.1103 (RStudio Team, 2021). The P-value < 0.05 was considered statistically significant.

## Results

### Study general characteristics

Totally, 70 preoperative patients (35 interventions and 35 placeboes) with a mean age of 53.13 ± 14.35 years assigned to the intervention and placebo groups from January to December 2020 (Fig. 1). In the intervention group, 2 subjects lost to follow-up and 3 of them discontinued to participate in the study. Also, 5 subjects in the placebo group were not interested to continue the participation in the study. Eventually, 60 subjects (30 in the Heallagen® and 30 in the placebo group) with more than 90% of compliance were analyzed. No adverse effect was observed following the Heallagen® or placebo consumption during the study. Thirty-six of subjects were male and 24 of them were female. As shown in Table 1, no significant difference was observed in age, weight, BMI, gender, and history of HTN between the two groups (p > 0.05).

**Table 1:** General characteristics of the participants according to the supplement or placebo groups

Variable	Heallagen (n = 30)	Placebo (n = 30)	P^a^
Age (years)	52.73 ± 15.59	53.53 ± 13.26	0.706*
Height (cm)	169.23 ± 9.69	168.47 ± 8.40	0.319
Weight (kg)	71.63 ± 8.95	73.87 ± 8.23	0.772*
Body mass index (kg/m^2^)	25.25 ± 4.34	26.30 ± 4.61	0.364
Gender			
Male	21 (70.0)	15 (50.0)	0.114
Female	9 (30.0)	15 (50.0)	
Hypertension history			
Yes	0 (0.0)	3 (10.0)	0.236
No	30 (100.0)	27 (90.0)	

Data are presented as mean ± SD for quantitative variables and frequency (%) for qualitative variables

^a^Calculated using independent sample t-test or Mann–Whitney U-test (indicated by *) for quantitative variables and Chi-square for qualitative variables

### Primary outcomes

#### Inflammatory markers

As shown in Table 2, the placebo group had a higher level of TNF-α and ESR at the baseline compared to the Heallagen® group (P = 0.010). No difference was observed between the two groups in other inflammatory markers at the beginning of the study. There was a significant increase in serum concentration of TNF-α, IL-6, hs-CRP, and ESR in both groups after surgery (P < 0.01). Supplementation with Heallagen® attenuated the increase in serum TNF-α, IL-6, hs-CRP and ESR levels compared to the placebo (P < 0.01). Also, the serum level of IL-1 was decreased following Heallagen® supplementation compared to the placebo (P < 0.001). After adjustments for the effect of BMI, age, gender, and history of hypertension, the findings remained unchanged.

**Table 2:** Comparison of the serum inflammatory markers between groups at the baseline and end of the study

Variable^a^		Heallagen (n = 30)	Placebo (n = 30)	P^b^	P-adjusted 1^c^
TNF-α(pg/ml)	Baseline	62.60 ± 40.56	86.63 ± 63.08	0.010*	0.011^¥^
	End of the study	142.93 ± 61.02	212.33 ± 196.37	0.745*	0.682^¥^
	Changes	80.33 ± 52.00	125.70 ± 196.44	0.865*	0.977^¥^
	P^d^	< 0.001*	< 0.001*		
IL-1(pg/ml)	Baseline	344.41 ± 17.55	340.21 ± 16.54	0.307*	0.380^¥^
	End of the study	289.23 ± 117.36	360.29 ± 169.66	0.062*	0.120^¥^
	Changes	− 55.17 ± 104.24	20.07 ± 109.57	0.032*	0.046^¥^
	P^d^	0.043*	0.504*		
IL-6(pg/ml)	Baseline	7.26 ± 2.81	6.44 ± 2.99	0.594*	0.366^¥^
	End of the study	10.67 ± 5.66	13.08 ± 5.55	0.023*	0.041^¥^
	Changes	3.41 ± 7.10	6.63 ± 7.44	0.370	0.108
	P^d^	< 0.001*	< 0.001*		
	P^d^	< 0.001*	< 0.001*		
ESR(mm/h)	Baseline	10.33 ± 11.02	21.56 ± 21.76	0.017*	0.046^¥^
	End of the study	17.50 ± 12.41	58.60 ± 33.78	< 0.001	< 0.001
	Changes	7.16 ± 11.77	37.03 ± 37.44	< 0.001	< 0.001
	P^d^	0.005*	< 0.001*		
hs-CRP(mg/l)	Baseline	2.54 ± 2.10	2.25 ± 0.79	0.388*	0.348^¥^
	End of the study	21.33 ± 11.54	51.06 ± 14.33	< 0.001*	< 0.001^¥^
	Changes	18.79 ± 11.16	48.81 ± 14.36	< 0.001	< 0.001
	P^d^	< 0.001*	< 0.001*		

*TNF-α* tumor necrosis factor α, *IL-1* interleukin-1, *IL-6* interleukin-6, *ESR* erythrocyte sedimentation rate, *hs-CRP* high sensitivity C-reactive protein

^a^Data are presented as mean (SD)

^b^Calculated using independent sample t-test or Mann–Whitney U-test (indicated by *)

^c^Calculated using ANCOVA or non-parametric ranked ANCOVA (indicated by ^¥^), adjusted for the effect of BMI, age, -gender and history of HTN

^d^Calculated using

paired t-test or Wilcoxon rank test (indicated by *)

### Secondary outcomes

As shown in Table 3, there was no significant difference in the counts of neutrophil, lymphocyte, platelets, WBC and the levels of BUN, and Creatinine between groups at the baseline (P > 0.05). Supplementation with Heallagen® resulted in a higher increase in neutrophil numbers compared to the placebo (P = 0.035). Also, the intervention group had a lower number of lymphocytes (P = 0.007) compared to the placebo at the end of the study. No difference was observed between groups in other variables. Adjustment for the effect of confounding variables did not change the results.

**Table 3:** Comparison of the hematological markers between groups at the baseline and end of the study

Variable^a^		Heallagen (n = 30)	Placebo (n = 30)	P^b^	P-adjusted 1^c^
Neutrophil (%)	Baseline	63.62 ± 7.28	65.4 ± 11.17	0.469	0.682
	End of the study	82.95 ± 9.29	79.62 ± 9.04	0.165	0.138
	Changes	19.32 ± 10.20	14.22 ± 13.83	0.035*	0.033^¥^
	P^d^	< 0.001	< 0.001		
Lymphocyte (%)	Baseline	30.51 ± 8.08	29.49 ± 10.53	0.677	0.811
	End of the study	11.54 ± 8.06	21.70 ± 20.69	0.007**	0.006**
	Changes	− 18.97 ± 9.68	− 7.79 ± 26.39	0.080*	0.085^¥^
	P^d^	< 0.001	0.117		
Platelet (10^3^/mm^3^)	Baseline	187.16 ± 41.90	196.43 ± 53.22	0.480**	0.770^¥^
	End of the study	164.46 ± 52.73	158.83 ± 29.64	0.410*	0.909^¥^
	Changes	− 22.70 ± 65.68	-37.60 ± 47.45	0.988*	0.949^¥^
	P^d^	0.057*	< 0.001*		
RBC (cells/mm^3^)	Baseline	4.85 ± 0.62	4.64 ± 0.65	0.536	0.702
	End of the study	3.67 ± 0.90	3.54 ± 0.73	0.728*	0.749^¥^
	Changes	− 1.17 ± 1.04	− 1.09 ± 0.84	0.765	0.812
	P^d^	< 0.001*	< 0.001*		
WBC (cells/mm^3^)	Baseline	6746.66 ± 1361.47	6834.00 ± 1903.15	0.839	0.714
	End of the study	11,421.33 ± 4760.87	10,122.16 ± 4124.20	0.459*	0.772^¥^
	Changes	4674.66 ± 4330.44	3288.16 ± 3817.91	0.379*	0.611^¥^
	P^d^	< 0.001*	< 0.001*		
BUN (mmol/L)	Baseline	16.83 ± 5.38	14.77 ± 4.29	0.107	0.112
	End of the study	16.59 ± 5.16	14.29 ± 3.74	0.064**	0.050**
	Changes	− 0.24 ± 4.88	− 0.48 ± 3.74	0.832	0.691
	P^d^	0.787	0.485		
Creatinine (mmol/L)	Baseline	1.28 ± 1.28	1.01 ± 0.24	0.255*	0.633^¥^
	End of the study	1.23 ± 1.31	1.44 ± 1.06	0.089*	0.091^¥^
	Changes	− 0.04 ± 0.28	0.43 ± 1.13	0.054*	0.208^¥^
	P^d^	0.402*	0.060*		

*RBC* red blood cells, *WBC* white blood cells, *BUN* blood urea nitrogen

^a^Data are presented as mean (SD)

^b^Calculated using independent sample t-test (variables that entered as logarithm form are indicated by **) or Mann–Whitney U-test (indicated by *)

^c^Calculated using ANCOVA (variables that entered as logarithm form are indicated by **) or non-parametric ranked ANCOVA (indicated by ^¥^), adjusted for the effect of BMI, age, -gender and history of HTN

^d^Calculated using paired t-test or Wilcoxon rank test (indicated by *)

## Discussion

During the 1-month of trial, consumption of 2 sachets/day daily of Heallagen® (a combination of 7 g Arg, 7 g Gln, and 1.5 g HMB) compared to the placebo led to a significant modulation in the serum levels of IL-6, hs-CRP, and ESR and significant reduction in the serum concentration of IL-1. Also, participants in the active treatment group had a significant higher platelet number and lower lymphocyte number than control group.

It has been reported in previous studies that serum levels of pro-inflammatory cytokines such as hs-CRP and TNF-α is closely related to the pathogenesis of atrial fibrillation (AF) [30] and compelling evidence shows that there is a link between inflammation and AF [31]. One of the common consequences after difficult surgeries, such as heart surgery, is systemic inflammatory response syndrome (SIRS). Inflammation is the body's response to tissue injury and is a rapid, highly amplified, controlled humoral and cellular response [32, 33]. Various factors are involved in exacerbating inflammation in patients after heart surgery, including stress, trauma, hypothermia, blood loss or transfusion and other factors. Also, it has been reported that some proinflammatory cytokines such as interleukin-1β and interleukin-6 play a critical role in the prediction of outcomes in critically ill patients [34]. The present study showed that at the end of the study, active treatment group had a slighter increase in IL-6, hs-CRP, and ESR concentration compared to the placebo group. Moreover, there was a significant reduction in the IL-6 in the intervention group compared to the placebo. TNF-α concentration was not significantly differed between placebo and active treatment groups at the end of trial.

In line with our findings, in a clinical trial Asjodi et al. showed that eight weeks supplementation with HMB led to a significant reduction in serum concentration of IL-6 and CRP after eccentric exercise in untrained males [35]. Also, Kraemer et al. reported a significant reduction in the serum levels of Interleukin-1β following HMB contained supplements. Interleukin-1β is a pro-inflammatory cytokine which plays an important role in the activation of T-cells in response to the antigen [36]. Higher concentration of the proinflammatory cytokines specially IL-1, IL-6, IL-8, and TNF-α have been showed in patients after heart surgery in several studies and can play a critical role in the multiple organ dysfunction syndrome (MODS) pathophysiology [37]. IL-6 is the major stimulator of acute-phase proteins generated by cardiovascular systems such as endothelial cells, vascular smooth muscle cells, and ischemic cardiomyocytes [38]. It has been demonstrated that the serum levels of IL-6 increase substantially in the first few days following heart surgery and then rapidly drops [39]. HMB's biological effects include changes in protein production, inhibition of proteolysis, and anti-inflammatory actions, which help to preserve muscle mass in both young and old subjects [40, 41]. Miyake and coworkers were evaluated the anti-inflammatory effects of HMB in TE-1 cancer cells and reported that HMB administration could downregulate IL-6 production by regulation of inhibitor of kappa B alpha (IĸBα) phosphorylation and reduction of nuclear translocation of NF-ĸB/Rela [42].

In addition to HMB, various studies indicated the anti-inflammatory effects of Gln and Arg. In an animal study Singleton et al. showed that Gln administration inhibits nuclear factor kappa-light-chain-enhancer of activated B cells (NF-kΒ) activation and cytokine expression in mice [43]. Moreover, Arutla et al. in a randomized clinical trial showed that Gln supplementation reduces the serum concentration of IL-6 among the patients with severe acute pancreatitis [44]. Various mechanisms have been proposed for the beneficial effects of Gln supplementation in patients with ischemic heart disease or undergoing heart surgery. Previously, it has been proposed that preserving myocardial glutamate throughout ischemia will help to produce high-energy phosphates by mitochondrial phosphorylation at the substrate level. Maintaining myocardial glutamate throughout ischemia and reperfusion is likely to lead to the provision of excessive α-ketoglutarate to the Krebs cycle, not only through alanine aminotransferase, also by creating a balance between α-ketoglutarate and glutamate [45, 46]. In addition, it was proposed that the preservation of intramyocardial glutamate throughout acute heart disease would cause glycolysis to continue without lactic acid accumulation but with alanine as the end result [16]. Furthermore, it has been reported in the study of Tostado et al. that Gln supplementation reduces myocardial damage after coronary revascularization under cardiopulmonary bypass [47]. It has been shown that part of the beneficial effects of Gln among the patients with heart surgery is attributed to the effect on Glutathione (GSH) and GSH scavenging properties [48, 49].

Also, Arg may exert its effects due to changes in immune cells. On the other hand, it has been shown that part of the anti-inflammatory effects of arginine is through modulation of the inflammatory responses induced by lipopolysaccharides (LPS). LPS, derived from gram-negative bacteria, is well known to activate innate immunity and induce an inflammatory response [50]. Qiu et al. in an animal study showed that arginine administration inhibited inflammatory pathways induced by LPS. On the other hand, the results of this study showed that arginine directly exerts inhibitory effects on the activity and expression of Toll-like receptor-4 (TLR4), Myeloid differentiation primary response 88 (MyD88), Cluster of differentiation 14 (CD14), NF-κB and IL-8 [51].

Moreover, in the present study, supplementation with a combination of Arg, Gln and HMB led to a significant increase in the platelet number and a significant reduction in the lymphocyte number. Rathmacher et al. showed that supplementation with a combination of these three amino acids leads to a significant improvement in the number of RBC, hemoglobin, hematocrit (HCT), lymphocytes, and eosinophils [52]. The precise mechanism behind these enhancements is unknown. One of the possible mechanisms is the effect of Arg deficiency on the maturation of blood cells and it has been reported that Arg deficiency can disrupt cell-mediated immune responses and mitogenic reactions in peripheral T cells [53]. Interestingly, previous studies have shown that supplementation with none of the Gln, Arg or HMB alone has not been shown to affect hematologic parameters significantly [54–56]. However, a combination of these three amino acids in the previous trials led to a significant improvement in RBC volume, HCT and hemoglobin levels [52, 57]. In a systematic review study, which conducted by Asghari et al., the effectiveness of Gln supplementation in cancer patients was evaluated and shown that the daily use of (Arg/Gln/HMB) mixture could improve some hematological parameters [58].

Various factors can affect people's response to supplementation. One of the most important factors is the nutritional status of the person before and after surgery. Accordingly, preoperative nutrition risk should be assessed and individualized intervention to be done [59]. Also, the history of previous chronic disease may change the result of the operation [60]. Therefore, the correct definition of inclusion criteria in these patients is of particular importance. In the present study, we have tried to control the factors affecting the results of the study. Moreover, the effect of important baseline characteristics including BMI, age, gender and history of HTN were adjusted. However, due to the limitations of the study, it was not possible to assess the nutritional status of individuals.

The present study had several strengths. This study shows that the supplementation of GLN, Arg, and HMB how can the clinical outcomes of surgery in practice. This study can be generalized to more population groups because all cardiac surgery patients were included and were not limited to a specific type of surgery. Based on our knowledge, the present study is the first clinical trial which was evaluated the effects of a combination of GLN, Arg, and HMB on clinical factors among the patients after heart surgery. Also, the appropriate duration of intervention and strong methodology were other strengths of this study. However, the present study has some potential limitations. Our sample size was relatively small, and we may have been underpowered to detect clinically relevant differences in biomarkers or clinical outcomes. Moreover, it is not clear which amino acids resulted these outcomes due to lack of groups with single-nutrient supplementation. Also, due to budget problems, it was not possible to assess the plasma levels of amino acids, especially glutamine.

## Conclusion

In conclusion, the results of the present study showed that a combination of Arg/Gln/HMB was able to reduce serum concentration of some inflammatory factors and hematological parameters. Further investigation with different dosages and longer intervention time may be warranted to determine the efficacy of a combination of HMB, Gln and Arg in patients with a heart surgery.

## Data Availability

The datasets used and/or analysed during the current study are available from the corresponding author on reasonable request.

## Abbreviations

GLN: Glutamine
HMB: β-Hydroxy-β-methylbutyrate
Arg: Arginine
TNF-α: Tumor necrosis factor-alpha
IL-1: Interleukin-1
IL-6: Interleukin-6
IL-10: Interleukin-10
BMI: Body mass index
HTN: Hypertension
ESR: Erythrocyte sedimentation rate
RBC: Red blood cells
WBC: White blood cells
BUN: Blood urea nitrogen
EDTA: Ethylene diamine tetra acetic acid
ICSH: International Council for Standardization in Hematology
ELISA: Enzyme-linked immunosorbent assay
ANCOVA: Analysis of covariance
AF: Atrial fibrillation
SIRS: Systemic inflammatory response syndrome
MODS: Multiple organ dysfunction syndrome
NF-κB: Nuclear factor kappa-light-chain-enhancer of activated B cells
IĸBα: Inhibitor of kappa B alpha
GSH: Glutathione
HCT: Hematocrit
